# A novel 3D-printed locking cage for anterior atlantoaxial fixation and fusion: case report and in vitro biomechanical evaluation

**DOI:** 10.1186/s12891-021-03987-2

**Published:** 2021-01-29

**Authors:** Shenglin Wang, Huijie Leng, Yinglun Tian, Nanfang Xu, Zhongjun Liu

**Affiliations:** Department of Orthopaedics, Peking University Third Hospital, Beijing, People’s Republic of China

**Keywords:** Irreducible atlantoaxial dislocation, 3D-printed implant, Patient-specific implants, Range of motion

## Abstract

**Background:**

Treatment of atlantoaxial dislocation is aimed at reduction and stabilization of the atlantoaxial joint. 3D printing refers to a process where additive manufacturing is achieved under precise computer control. Literature on its utilization in anterior atlantoaxial fixation and fusion is rare. This study is the first report on a 3D-printed locking cage used in the anterior procedure for atlantoaxial dislocation.

**Methods:**

A middle-aged male in his 40s presented with weakness and numbness of his extremities for 3 years and could only walk slowly with assistance. Imaging studies revealed severe anterior migration of C1, irreducible atlantoaxial dislocation, and severe cervical-medullary compression. A preoperative plan consisting of trans-oral soft tissue release and fixation using tailor-designed 3D-printed cages was devised. Following fluoroscopic confirmation of reduction of the atlantoaxial joints, two customized 3D-printed cages made of titanium alloy were inserted into the bilateral facet joints, which were then locked by six screws into the lateral masses of C1 and C2. The microstructure of the inserted cages was optimized for improved biomechanical stability and enhanced osseo-integration, without the need for bone grafting. In addition, a biomechanical test was performed on seven human cadaveric specimens comparing the novel implant with the conventional C1 lateral mass-C2 pedicle screw construct in three modes of motion (flexion-extension, lateral bending, axial rotation).

**Results:**

Improvement of neurologic function in the patient was evident immediately after surgery. He was able to walk independently 1 month post-operatively. At the 12-month follow-up, coronal reconstruction of CT demonstrated properly-positioned 3D-printed cages, evidence of osseo-integration at the bone-implant interface, and no subsidence or displacement of the implant. Eighteen months out of surgery, the mJOA score improved to 15, and lateral X-ray confirmed reduction of atlanto-axial dislocation. Additionally, the new construct provided strong fixation comparable to that conferred by conventional constructs as there was no significant difference observed between the two groups in all three directions of motion.

**Conclusions:**

The novel implant represents a new option in the treatment of irreducible atlantoaxial dislocation. It can provide strong anterior support for solid fixation and fusion with a low profile and a microstructure that obviates the need for bone grafting.

**Supplementary Information:**

The online version contains supplementary material available at 10.1186/s12891-021-03987-2.

## Background

In the management of irreducible atlantoaxial dislocation (IAAD), the goal of treatment consists of restoration of the normal anatomy of the cranio-vertebral junction (CVJ), complete decompression of the lower medulla and the upper spinal cord, and stabilization of the atlantoaxial joints [1, 2]. The main procedures reported in the literature include trans-oral odontoidectomy or peri-odontoid tissue release followed by posterior occipito-cervical or atlanto-axial fusion [3–5]. Anterior-only (with peri-odontoid soft tissue release followed by anterior fixation) represents another strategy which eliminates the need for a two-step operation [6]. This single-stage procedure used a reduction plate to achieve decompression, reduction and fusion, all through a trans-oral incision. However, the trans-oral reduction plate had a high profile and still required bone harvest from the iliac crest [6], as did the two-stage procedures. Although there had been attempts on using rapid prototyping (3D printing) to facilitate instrumentation in the upper cervical spine [7, 8], to our knowledge, its utilization in anterior atlantoaxial fixation has not been reported. We herein describe the use of a novel self-locking implant with semi-constrained screws in a patient with IAAD. In addition, the biomechanics of this new 3D-printed locking cage (3DPLC) was evaluated in seven cadaver specimens.

## Methods

### Clinical report

**Fig. 1:**
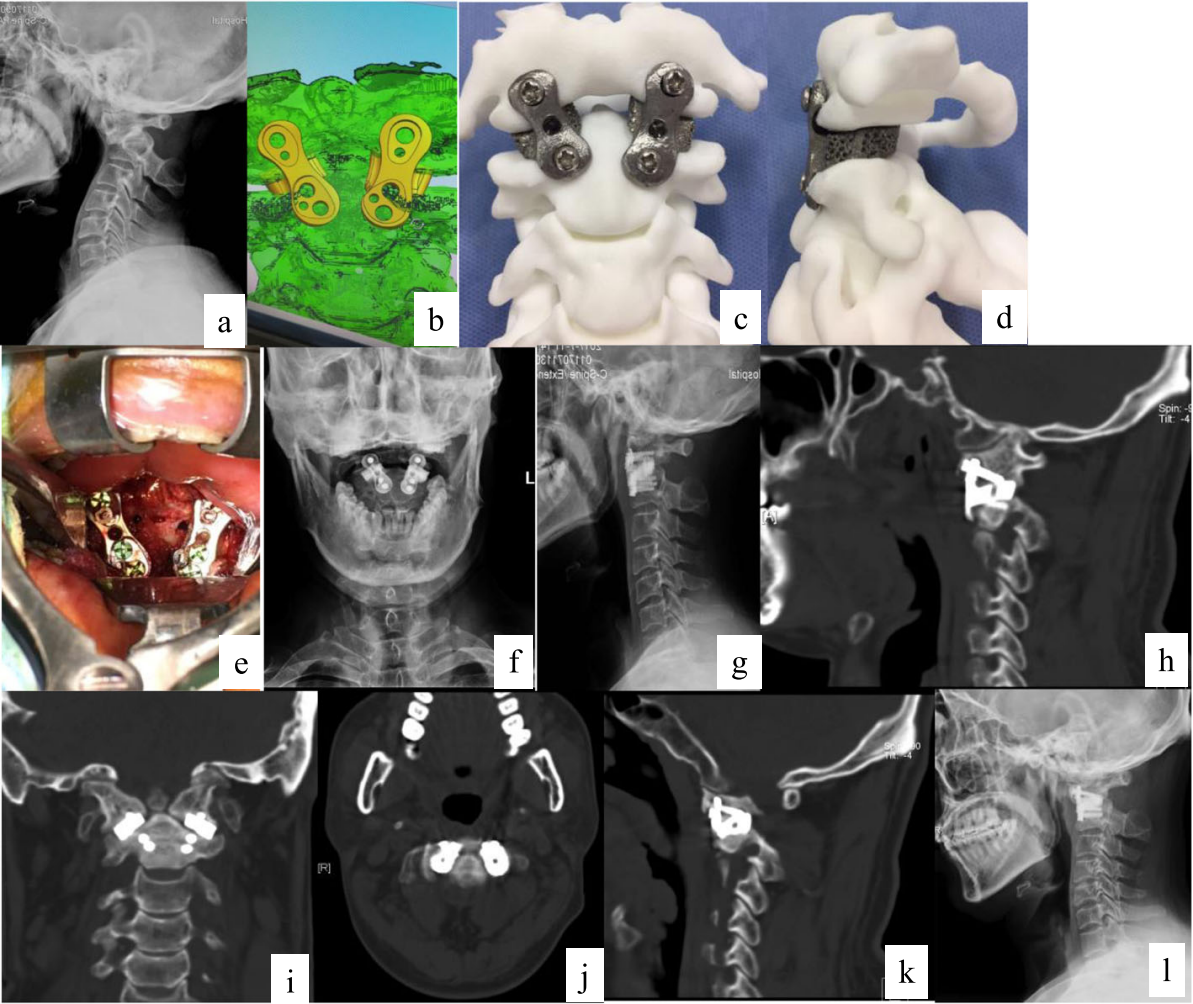
**a** Pre-operatively lateral X-ray; **b** Anterior view of the computer-aided design model of 3DPLC; **c** Anterior view of the manufactured 3DPLC based on 1b on a 3D-printed model; **d** Lateral view of the manufactured 3DPLC based on 1b on a 3D-printed model; **e** Intra-operative view of the 2 3DPLC before closure; **f** Anteroposterior X-ray 1 week after the surgery; **g** Lateral X-ray 1 week after the surgery; **h** Left sagittal reconstruction of CT of the cervical spine 12 months post-operatively; **i** Coronal reconstruction of CT of the cervical spine 12 months post-operatively; **j** Axial CT of the cervical spine 12 months post-operatively; **k** Right sagittal reconstruction of CT of the cervical spine 12 months post-operatively; **l** Lateral X-ray 18 months after the surgery

A 44-year-old man presented with weakness and numbness of both upper and lower extremities for 3 years and had no history of cervical trauma. He could ambulate using a walker and had deficits of motor function in the right hand. His mJOA (modified Japanese Orthopaedic Association, supplementary file) [9] score was 11. Sensory dysfunction of the upper extremities was detected upon neurological examination and symmetric hyperreflexia was present in the lower extremities. Positive Hoffmann’s signs were elicited bilaterally. His gait was wide-based, spastic, and unsteady. The patient also had limited cervical flexion and extension. Imaging studies revealed AAD with severe anterior translation of C1 on X-ray (Fig. 1a) and significant cervical-medullary cord compression with high signal intensity on T2 MRI. He was diagnosed of IAAD, and as it was not reduced with a 1/5 body weight cranial traction, trans-oral peri-odontoid release was required to allow for anatomic reduction [5]. Additionally, in order to obviate an additional posterior procedure to provide fixation and fusion, with informed consent from the patient, we decided to use a novel implant, the 3DPLC, to perform an anterior-only surgery. The 3DPLC was manufactured using titanium alloy powder based on a pre-designed computer model customized to the specific anatomy of the C1-C2 facet joint of this particular patient (Fig. 1b,c, d, and e). The model was built from a thin-cut CT scan of the upper cervical spine using Materialise Interactive Medical Image Control System (MIMICS). During the procedure, complete reduction was first achieved following peri-odontoid soft tissue release, and the two tailor-fabricated 3DPLCs were implanted into the C1-C2 facet joints. Position of the cages were confirmed by C-arm image intensification, and three screws were used (one on C1 lateral mass and two on C2 vertebral body) to lock each cage (Fig. 1f, g and h). Use of this novel implant was approved by the local hospital ethics committee.

### Biomechanical evaluation

#### Specimen preparation

**Fig. 2:**
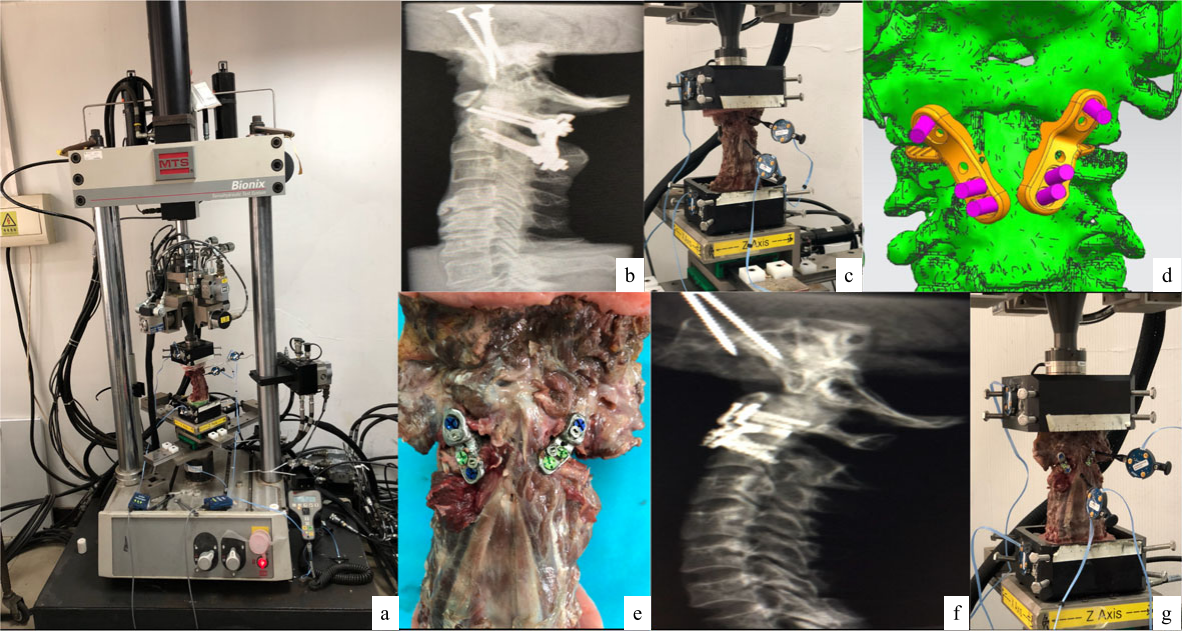
**a** Overview of the specimen and the spine motion simulation system; **b** Lateral X-ray after posterior instrumentation; **c** Setup for biomechanical evaluation following posterior instrumentation; **d** Computer-aided design model of 3DPLC; **e** Specimen with 3DPLC inserted anteriorly; **f** Lateral X-ray after anterior instrumentation; **g** Setup for biomechanical evaluation following anterior instrumentation

Seven fresh frozen human cadaveric cervical spine specimens (kept at − 20 degrees in sealed bags) from subjects with no history of trauma or deformities involving the cervical spine were used (Fig. 2a). No specimen was osteoporotic as confirmed by densitometry. Soft tissue structures, including the ligaments, joint capsules, intervertebral discs were all intact on visual inspection. Before the test, specimens were thawed overnight at room temperature. Neutral alignment of the cervical spine was carefully maintained during preparation when both ends of the specimen were embedded with polymethylmethacrylate (PMMA) and mounted onto a spine motion simulator (MTS Corp, Eden Prairie, MN USA).

#### Testing protocol

Pilot markers were attached anteriorly to the C1 right lateral mass and the C2 vertebral body to track their respective segmental range of motion (ROM), which was measured and analyzed using NDI Optotrak Certus, a 3D movement measuring system (Norther Digital Inc., Ontario, Canada). Classic posterior C1 lateral mass-C2 pedicle screw (C1LM-C2PS) instrumentation [10] was first performed. Each specimen was pre-conditioned for three loading cycles with moment variation between 2 Nm and − 2 Nm to minimize viscoelasticity effects. (The moment of 2 Nm was chosen because based on the literature [9, 11], the most commonly used values in cervical spine studies range from 1.5 Nm to 2.5 Nm.) Then, starting from a neutral position, each specimen was tested in three different directions of motion in the order of extension-flexion (i.e. anterior-posterior translation), lateral bending, and axial rotation, while keeping the displacement under control between angles corresponding to the moments between 2 NM and -2NM as determined in the previous viscoelasticity mitigation step (Fig. 2b, c). The posterior implants were then removed and two personalized 3DPLCs were inserted anteriorly between the C1-C2 facet joints (Fig. 2d, e, f, g) in each specimen. A second round of biomechanical evaluation was then performed. Segmental movement between C1 and C2 was measured for each direction of motion with either type of internal fixation and data were analyzed using paired sample T-test (SPSS, 17.0 statistics). The precision of the NDI system for rigid body displacement measurement is 0.01 mm.

## Results

### Clinical results

Improvement of his neurological function was evident immediately after surgery. A Philadelphia collar was used for 3 months for outdoor activities. He was able to walk independently 1 month post-operatively. At the 12-month follow-up, CT scan (axial, sagittal, coronal) demonstrated properly-positioned 3DPLCs, evidence of osseo-integration at the bone-implant interface, and no subsidence or displacement of the 3DPLCs (Fig. 1i, j, k, and l). Eighteen months out of surgery, the mJOA score improved to 15, and lateral X-ray confirmed reduction of atlanto-axial dislocation. Even though partial osseous collapse of C1 lateral masses was suspected, there was no implant subsidence or loosening of the screws (Fig. 1m).

### Biomechanical results

**Fig. 3:**
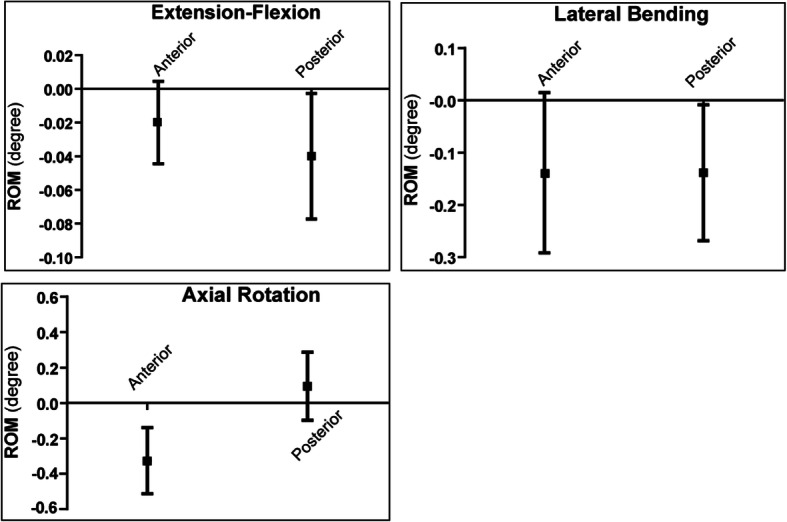
In vitro biomechanical tests showed that segmental movement in all three directions was limited and there was no significant difference between the two types of fixation

Both the posterior and the anterior constructs provided strong fixation. In all three directions of motion, the amount of segmental movement was limited and there was no significant difference observed between the two types of fixation (Fig. 3, Table 1).

**Table 1:** Comparison between 3DPLC and the conventionl posterior construct in three modes of motion

Motion mode	ROM (degree)		
	Anterior Fixation (Mean ± SEM)	Posterior Fixation (Mean ± SEM)	*P* value
Flexion & Extension	0.024 ± 0.028	−0.004 ± 0.005	0.33
Lateral Bending	−0.013 ± 0.006	0.017 ± 0.015	0.09
Axial Rotation	−0.007 ± 0.003	−0.173 ± 0.167	0.34

## Discussion

We previously described the first utilization of 3D-printed implants in spine surgery in 2016 for anterior C2 reconstruction following spondylectomy due to Ewing sarcoma in an adolescent [8]. Phan et al. [12] reported a posterior 3D-printed implant for atlanto-axial arthrodesis in a 65-year female, however, she was followed up for only 2 months. Additional report on the use of 3D-printed constructs in the upper cervical spine was rare in the literature.

The purpose of the current study was to illustrate the first anterior customized implant (3DPLC) in the upper cervical spine for an IAAD patient with mid-term follow-up. Surgical treatment of IAAD consisted of two steps: release of the peri-odontoid soft tissue to allow anatomic reduction (typically trans-oral from the anterior) and strong fixation (typically from the posterior) to allow early fusion. 3DPLC was an anterior implant that could provide solid fixation (comparable to the strongest posterior construct in all forms of ROM) along with a cage that facilitated osseo-integration. Since trans-oral release often needed to be performed in patients with IAAD, any posterior instrumentation would require a second surgical procedure with associated morbidity, which could have been avoided if an anterior instrumentation and fusion procedure were to be performed following trans-oral release. Use of our novel implant following the trans-oral release procedure could help avoid both a second-stage posterior surgery and the need for iliac bone harvesting. Additionally, it could be used as a salvage technique in cases where abnormal anatomy or previous surgical procedures precluded the use of posterior instrumentation and anterior instrumentation became the only viable option.

Existing posterior constructs for atlantoaxial fixation included C1LM–C2PS, C1-C2 trans-articular screws (C1–C2 TA), C1 lateral mass and C2 trans-laminar screws (C1LM-C2TL), and C1-C2 lateral mass screws (C1–C2 LM). The biomechanical properties of these constructs had been assessed in previous studies [13–15]. A meta-analysis [13] of fifteen articles found that C1-C2 LM is less stable against axial rotation, C1LM–C2TL is less stable against lateral bending, while C1LM–C2PS represents the strongest form of atlantoaxial fixation. On the other hand, the primary form of anterior fixation in the atlantoaxial region in the literature was the anterior trans-oral atlantoaxial reduction plate (TARP). The TARP construct offered comparable stability to the C1LM-C2PS constructs in all directions of segmental movement except for flexion [16]. In comparison, results from the current study showed that 3DPLC could provide stability in all directions of movement equivalent to the conventional C1LM–C2PS construct (Fig. 3). Additionally, fixation of the new 3DPLC was easier than the TARP construct and its 3D-printed porous micro-structure could facilitate osseo-integration, such that graft harvest from the iliac crest, which could be fraught with complications (e.g. pain, wound dehiscence, etc.), became obsolete with this new implant. Lastly, the 3DPLC had a lower profile when compared to TARP, especially in the midline where the incision was made. Therefore, the 3DPLC might better facilitate wound healing in the posterior pharyngeal wall.

There were several limitations to this study. First, this was a case report with mid-term follow-up; second, the biomechanical evaluation did not include a fatigue test; and third, it currently took 2-3 weeks to manufacture the customized implant, prohibiting its use in emergency cases. However, developing anterior implants for atlantoaxial instrumentation and fusion was a project worthy of continuing efforts and could be built upon results from our current study.

## Conclusions

The novel implant herein described represented a new option in the treatment of irreducible atlantoaxial dislocation that could provide strong anterior support for solid fixation and fusion with a low profile and a microstructure that obviated the need for bone grafting.

## Supplementary Information

**Additional file 1.**

## Data Availability

Available upon reasonable request to the corresponding author.

## Abbreviations

mJOA: modified Japanese Orthopaedic Association
ROM: range of motion
IAAD: irreducible atlantoaxial dislocation
CVJ: cranio-vertebral junction
3DPLC: 3D-printed locking cage
AAD: atlantoaxial dislocation
PMMA: polymethylmethacrylate
C1LM-C2PS: C1 lateral mass-C2 pedicle screw
C1–C2 TA: C1-C2 trans-articular screws
C1LM-C2TL: C1 lateral mass and C2 trans-laminar screws
C1–C2 LM: C1-C2 lateral mass screws
TARP: trans-oral atlantoaxial reduction plate
