# Supplementary figures and images for: A novel 3D-printed locking cage for anterior atlantoaxial fixation and fusion: case report and in vitro biomechanical evaluation
Source: BMC Musculoskelet Disord. 2021 Jan 29;22:121. doi: 10.1186/s12891-021-03987-2 (PMC7844893; doi:10.1186/s12891-021-03987-2)

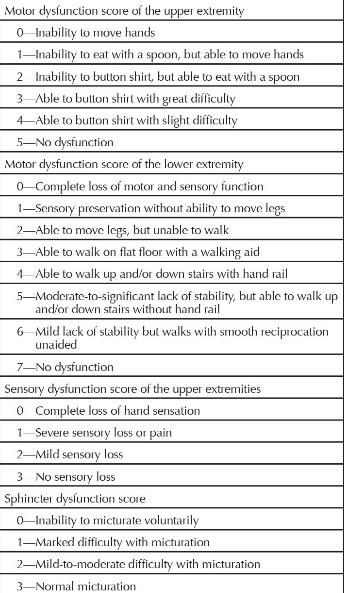

Supplement: Supplementary file 1 — Additional file 1. [file 12891_2021_3987_MOESM1_ESM.jpg]
